# Breast cancer incidence, stage distribution, and treatment shifts during the 2020 COVID-19 pandemic: a nationwide population-level study

**DOI:** 10.1186/s13690-024-01296-3

**Published:** 2024-05-07

**Authors:** Hanna M. Peacock, Lien van Walle, Geert Silversmit, Patrick Neven, Sileny N. Han, Nancy Van Damme

**Affiliations:** 1 Research Department, Belgian Cancer Registry, Koningsstraat 215 bus 7, Brussels, 1210 Belgium; 2 Department of Gynecological Oncology and Multidisciplinary Breast Centre, University Hospitals Leuven, Leuven, Belgium

**Keywords:** COVID-19 pandemic, Breast Cancer, Diagnostic Delay, TNM Stage, Population-based data, Cancer Registry, National Healthcare

## Abstract

**Background:**

The first COVID-19 wave in 2020 necessitated temporary suspension of non-essential medical services including organized cancer screening programs in Belgium. This study assessed the impact of the pandemic on breast cancer (BC) incidence, stage at diagnosis, and management in Belgium in 2020.

**Methods:**

All Belgian residents diagnosed with in situ or invasive BC in 2015–2020 in the nationwide, population-based cancer registry database were included. Incidence trends for 2015–2019 were extrapolated to predict incidence and stage distribution for 2020 and compared with the observed values. National healthcare reimbursement data were used to examine treatment strategies. Exact tumor diameter and nodal involvement, extracted from pathology reports, were analyzed for 2019 and 2020.

**Results:**

74,975 tumors were selected for analysis of incidence and clinical stage. Invasive BC incidence declined by -5.0% in 2020, with a drop during the first COVID-19 wave (Mar-Jun; -23%) followed by a rebound (Jul-Dec; +7%). Predicted and observed incidence (in situ + invasive) was not different in patients < 50 years. In the 50–69 and 70 + age groups, significant declines of -4.1% and − 8.4% respectively were found. Excess declines were seen in clinical stage 0 and I in Mar-Jun, without excess increases in clinical stage II-IV tumors in Jul-Dec. There was no increase in average tumor diameter or nodal involvement in 2020. Patients diagnosed in Mar-Jun received significantly more neoadjuvant therapy, particularly neoadjuvant hormonal therapy for patients with clinical stage I-II BC.

**Conclusions:**

BC incidence decline in 2020 in Belgium was largely restricted to very early-stage BC and patients aged 50 and over. Delayed diagnosis did not result in an overall progression to higher stage at diagnosis in 2020. Observed treatment adaptations in Belgium were successful in prioritizing patients for surgery while preventing tumor progression in those with surgical delay. Continuation of monitoring BC incidence and stage in the future is crucial.

**Supplementary Information:**

The online version contains supplementary material available at 10.1186/s13690-024-01296-3.

**Table:** 

**Text box 1. Contributions to the literature**
• Assessing the effect of the COVID-19 pandemic on breast cancer incidence, stage at diagnosis and treatment must consider population-level historical trends
• Incidence of early-stage breast cancer was lower in the first wave of the pandemic but recovered in the second half of 2020 in Belgium
• At the national level, no shift to more advanced stage at diagnosis was observed during the second half of 2020 in Belgium
• Treatment adaptations, such as increased use of neoadjuvant hormonal therapy to delay surgery, were appropriately applied

## Background

The global pandemic of coronavirus disease 2019 (COVID-19) has impacted healthcare worldwide. The first wave of the COVID-19 pandemic in Belgium took place from 1 March – 22 June 2020, lockdown was initiated on 14 March 2020, and all “non-essential” healthcare, including organized population screening for breast, cervical and colorectal cancer, was temporarily halted [1, 2].

The Belgian healthcare system is based on compulsory health insurance [3]. The organization of cancer screening is a regional responsibility and breast cancer screening programs exist for women aged 50–69 [4, 5]. Overall, breast cancer screening coverage (organized and opportunistic screening mammograms) in Belgium lies around 60% [6]. Due to COVID-19, breast cancer screening was halted mid-March 2020 and resumed by end June 2020 in Belgium [2].

Female breast cancer (BC) is the most frequently occurring cancer type in Belgium [7]. A first estimate of cancer incidence in 2020 based on accelerated pathology reporting estimated a -50% decline in diagnosis of invasive BC among females in April 2020, with 6% of diagnoses for female BC still outstanding at the end of 2020 [8].

To ensure optimal cancer treatment within the context of a healthcare system overwhelmed by COVID-19, treatment alterations were recommended by several multidisciplinary panels [9–13]. In EUSOMA (European Society of Breast Cancer Specialists) certified breast centers, neoadjuvant systemic treatment was used to postpone surgery as safely as possible; neoadjuvant hormonal therapy, chemotherapy and targeted therapy indications were modified in 23%, 23% and 10% of centers, respectively [14].

The current study aimed to evaluate absolute incidence, stage at diagnosis, and initial treatment of BC covering the entire population of Belgium in 2020, by comparing the observations with predictions based on temporal trends over the previous five years.

## Methods

### Methods - data sources

Reporting of all cancer diagnoses to the Belgian Cancer Registry (BCR) by both oncological care programs and pathology laboratories is mandatory in Belgium [15]. The available BCR data consist of clinical information (patient characteristics: date of birth, sex assigned at birth, place of residence, WHO performance score [16]; and tumor characteristics: incidence date, Union for International Cancer Control (UICC) cTNM and pTNM classification [17, 18], topography code [19], grade, behavior, laterality, and morphology code [20]), supplemented with pathology text-protocols received from the pathology laboratories.

For operated invasive BC for which the data was available, in incidence years 2019 and 2020, detailed information on pathological tumor diameter (*N*(2019) = 8625 tumors and *N*(2020) = 9064 tumors) and lymph node involvement (*N*(2019) = 8490 tumors and *N*(2020) = 7747 tumors) was manually extracted from the pathology protocols (Supplemental Table 1).

The patient’s national social security number enables linkage with national health insurance data from the Intermutualistic Agency (IMA) to obtain details on reimbursed diagnostic and therapeutic procedures as well as pharmaceuticals (follow-up until 30 June 2021). These linkages have been approved by the former Belgian Privacy Commission [21, 22]. IMA-data are linked to a unique patient, but not to a specific diagnosis, therefore only procedures and pharmaceuticals administered within a defined period around the incidence are considered. Diagnostic procedures were retained from IMA-data in the − 90/+90 days interval around incidence. Surgery and, in the absence of surgery, radiotherapy and systemic treatment (ST) were included − 30/+270 days around incidence. Neoadjuvant therapy (NAT) was captured from 30 days before incidence to the day before surgery. Adjuvant therapy was captured from the day of surgery to 270 days after surgery.

### Data availability statement

The cancer cohort data used and analyzed during the study are available from the corresponding author upon reasonable request. The pseudonymized data can be provided within the secured environment of the BCR after having been guaranteed that the applicable GDPR regulations are adopted.

### Methods - study population

All Belgian residents (male/female) with an in situ or invasive breast tumor diagnosed in the period 2015–2020 were selected from the BCR database (*N* = 78,761). Sarcoma, Paget’s disease and in situ tumors other than ductal and lobular in situ carcinoma (D/LCIS) were excluded (*N* = 752). To enable linkage to the IMA database, patients without Belgian residency (*N* = 1197), patients without national social security number (*N* = 159) and patients for whom linkage could not be made to IMA-data (*N* = 1,678) were excluded. The final study population used to evaluate tumor incidence and stage is provided in Supplemental Table 2 (*N* = 74,975). For the analyses of diagnosis and treatment, patients with another invasive tumor or breast tumor (in situ or invasive) in 2004–2020 were excluded (*N* = 14,296). Due to small numbers, detailed trend analysis of diagnostic/therapeutic procedures for in situ tumors was not possible.

Incidence date is defined as the date of microscopic confirmation of malignancy. If unavailable, in descending order of priority, the date of first hospitalization/consultation for malignancy, first technical examination, start of treatment, or date of death is used. For the analyses by tumor stage, the clinical TNM stage is used, given that the initial treatment is guided by the clinical stage [17, 18]. Comorbidities are calculated based on reimbursed medication in the year before incidence [23].

Based on preceding explorations of completeness, the study period for the treatment analyses was limited to January-September 2020 to ensure completeness of reimbursement data.

### Methods - statistical analyses

#### Predicted numbers

A Poisson count model was used to generate the predicted value (*N*_*pred(tot)*_) of expected overall number of BC diagnoses in 2020 [24]. The observed count was taken as the dependent variable and incidence year as the independent variable in the Poisson model. The SAS 9.4 *proc genmod* procedure with an identity link was used to estimate the absolute average yearly change in the number of BC diagnoses over the period 2015–2019. Overdispersion was not considered. This linear model was extrapolated to 2020 and used as expected count for 2020 (Supplemental Fig. 1A). The models were done for invasive BC, DCIS and LCIS separately. Three phases of the pandemic in 2020 were examined and compared to the trends in the same time periods in 2015–2019: January-February (pre-pandemic), March-June (first wave of the pandemic in Belgium), and July-December (recovery period: cancer incidence generally returned to baseline from June 2020 despite the start of the second wave of the pandemic in Belgium 31 August 2020) [1, 8]. Exact confidence intervals (CIs) for the observed values were calculated using the method of Daly [25]. The difference between the predicted and the observed number of diagnoses (*N*_*diff*_; Supplemental Fig. 1) was considered significant if the 95%CI of the difference did not contain zero.

Similarly, predicted values were established for specific subgroups (for stage groups, treatment schemes). The obtained individual predicted values (*N*_*pred*_) were corrected for the overall observed percent decline in diagnosis for the total population, to produce an individual corrected-predicted value (*N*_*pred(corr)*_) for each subgroup (Supplemental Fig. 1B). For example, to assess shifts in tumor stage at diagnosis during the year 2020, we repeated the Poisson count model predictions for each clinical stage (cStage) independently and corrected for the total decline in incidence. For the subgroup tumors with unknown cStage, due to an abrupt improvement in completeness of stage registration in 2017, the period 2017–2019 was used as the reference to permit a linear trend.

#### Other statistical analyses

The Pearson chi square test was used to explore differences in distribution of categorized patient/tumor characteristics in 2020 versus 2015–2019 (Supplemental Tables 2–4). For mean age and mean time to treatment, a standard t-test was used if variances were equal according to Levene’s test for Homogeneity of Variance. If variances were significantly different, the Welch’s t-test was used. For comparisons of mean tumor diameter and mean number of positive lymph nodes, a Wilcoxon Z-Test was used. The 95% CIs are reported and obtained from the normal approximation unless otherwise stated.

## Results

### Breast cancer incidence in 2020

#### Decline in diagnosis of BC during the first wave of the COVID-19 pandemic, followed by a partial recovery

In total 74,975 breast tumors were selected for analysis of incidence and stage at diagnosis. For patient and tumor characteristics see Supplemental Tables 2–4.

**Fig. 1:**
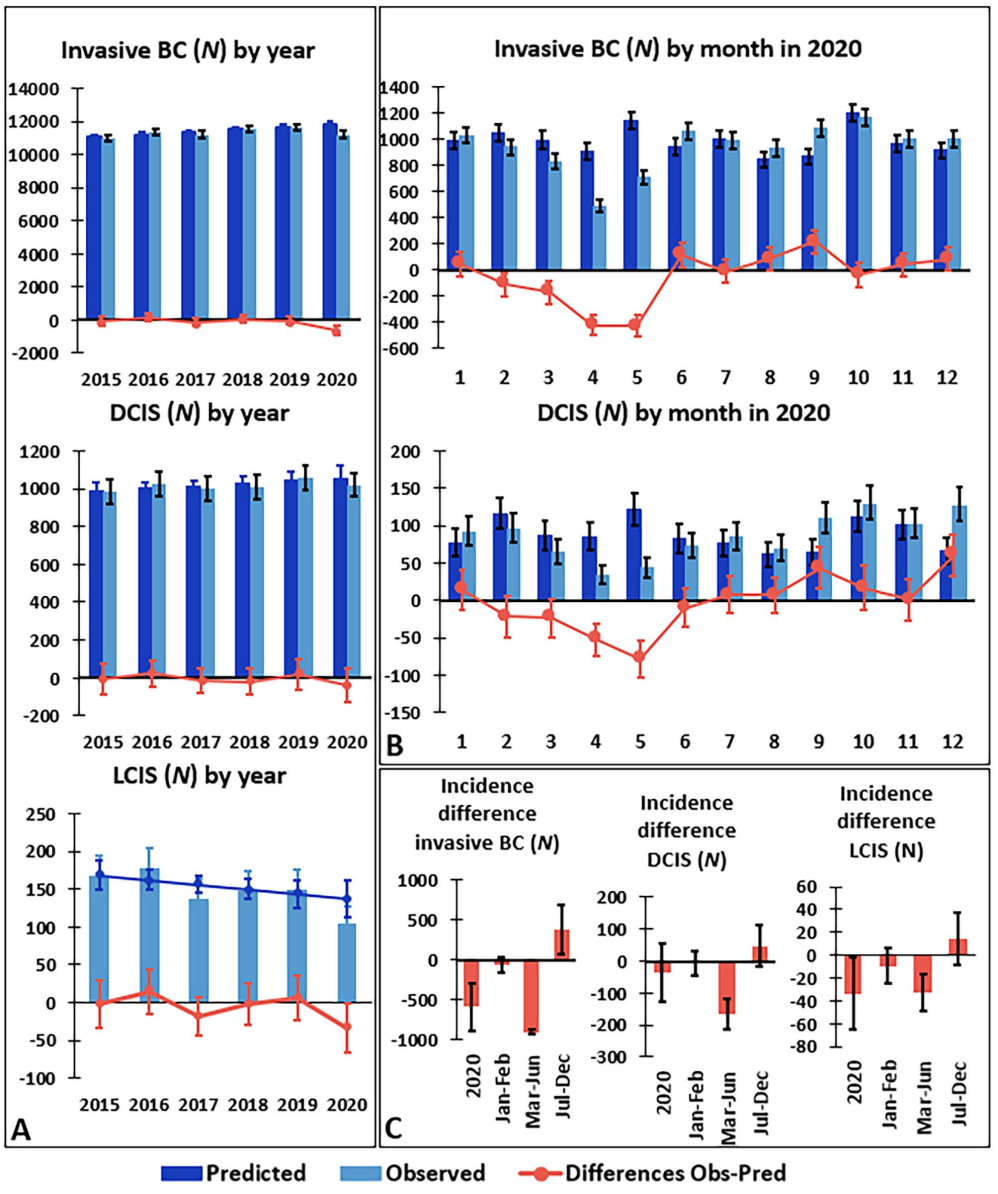
Total number of breast cancer diagnoses declined in 2020. Predicted (dark blue) and observed (light blue) number of cases, and the difference between predicted and observed (orange line) of invasive BC, DCIS and LCIS by year (**A**) and by month in 2020 (**B**; no data for LCIS because of low numbers). Difference between observed and predicted number of cases of invasive BC, DCIS and LCIS by pandemic phase in 2020 (**C**). Error bars represent 95% confidence intervals. Differences between observed and predicted number of cases are significant if the 95% confidence interval does not contain 0. BC = Breast cancer. DCIS = Ductal carcinoma in situ. LCIS = Lobular carcinoma in situ

Absolute incidence of invasive BC (*N*(2015) = 10,993; *N*(2019) = 11,648) and DCIS (*N*(2015) = 983; *N*(2019) = 1,058) increased between 2015 and 2019 but declined in 2020 (BC *N* = 11,216, DCIS *N* = 1,018). For invasive BC there was a significant decline in observed incidence in 2020 of 590 diagnoses (95%CI [287,893]; -5.0%) compared to the predicted value (Fig. 1A&C). Compared to monthly projections for 2020, there was a significant decline in invasive BC cases during the first wave of the pandemic (March-June) of -23%, with a maximal monthly decline of -47.9% in April 2020 (Fig. 1B&C). A significant increase in incidence of 7% was observed in July-December. For DCIS there was a significant decline of -44% in March-June, with a maximal monthly decline of -64.8% in May, while a significant increase was observed in September and December (Fig. 1A, B&C). For LCIS, a significant decline in absolute incidence was observed in 2020; however, given the small numbers, no further sub-analyses were performed (Fig. 1A&C).

Total incidence (in situ + invasive) was examined by age group (< 50, 50–69, 70+; Supplemental Fig. 2A). The decline in observed incidence in 2020 was more pronounced with increasing age. No significant difference between predicted and observed incidence was found in the age group < 50 years. In the 50–69 and the 70 + age groups, significant declines of -4.1% and − 8.4% respectively were documented for 2020. In the 50–69 age group, a decline of 722 diagnoses (95%CI [602,842]; -34.1%) in March-June was found, followed by a partial recovery of 470 diagnoses (95%CI [311,629]; +16.1%) in July-December. By contrast, in the 70 + age group the decline in March-June was smaller (*N* = 326; 95%CI [222,430]; -21.0%) and no recovery was observed in July-December.

#### Decline in diagnosis of early-stage BC during the first wave of the pandemic did not result in more advanced-stage tumors in the recovery period in 2020

**Fig. 2:**
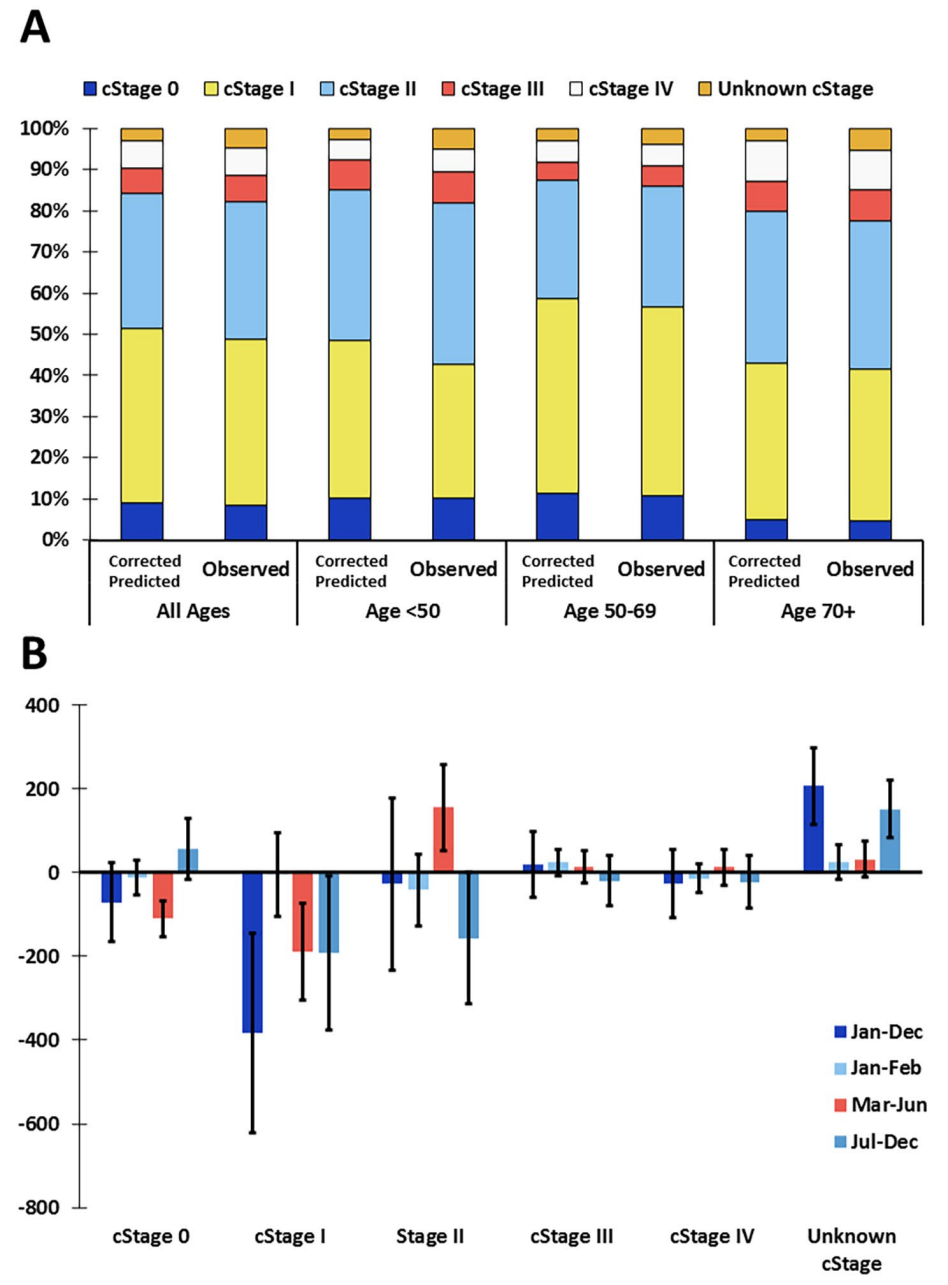
More pronounced decline in diagnosis in lower stage breast cancers. (**A**) Predicted (corrected for total decline in diagnoses) and observed clinical stage distributions in 2020 per age group indicating the percentage of cases (invasive or in situ) per stage. (**B**) Difference between the number of observed cases and the corrected-predicted number of cases of invasive or in situ breast cancer in 2020 by clinical stage and by month of incidence. Error bars represent 95% confidence intervals. When the 95% confidence interval does not contain 0, the observed number of cases is significantly different from what would be predicted if the numbers in all stages declined (or increased) proportional to the total decline (or increase) in cases relative to the reference period

The observed incidence numbers by cStage in 2020 were significantly lower than the corrected-predicted values for cStage 0 (in situ) tumors only in March-June, for cStage I tumors in March-June, July-December and over the whole of 2020 (Fig. 2A-B). For cStage II tumors, the observed number in March-June was significantly larger than the corrected-predicted number. During the recovery phase, July-December, there was no excess in tumors diagnosed with cStage II, III or IV.

The cStage distribution in 2020 varied by age group (Supplemental Fig. 3A-C). A significant decline in cStage 0 in March-June was only observed in the 50–69 age group. In the age groups < 50 years and 50–69 years, a decline in cStage I tumors was observed over the whole of 2020, in March-June and in July-December, and an increase of cStage II tumors was observed in March-June. In the 70 + age group, the observed declines in diagnosis by cStage were proportional to the total decline in incidence.

The observed number (*N* = 568) and percent (4.6%) of registrations with unknown cStage in 2020 were comparable to those observed in 2019 (*N* = 573, 4.5%).

#### Delayed diagnosis did not result in overall augmented tumor diameter or nodal involvement in 2020

No statistically significant differences were observed in average pathological tumor diameter or number of positive lymph nodes for all operated invasive tumors in 2020 compared to 2019 (Supplemental Table 1). The results were also compared specifically among patients with (2019 15–16%; 2020 14–17%) or without NAT, and also in the three time periods separately (Supplemental Tables 1 & Fig. 5A-D).

### Breast cancer management in 2020

#### No change in diagnostic approach

The number of patients undergoing various diagnostic examinations was not different from what was predicted, apart from increased usage of CT-scans of the body in 2020 (Supplemental Fig. 6).

#### More use of hormonal NAT for early-stage invasive BC

**Fig. 3:**
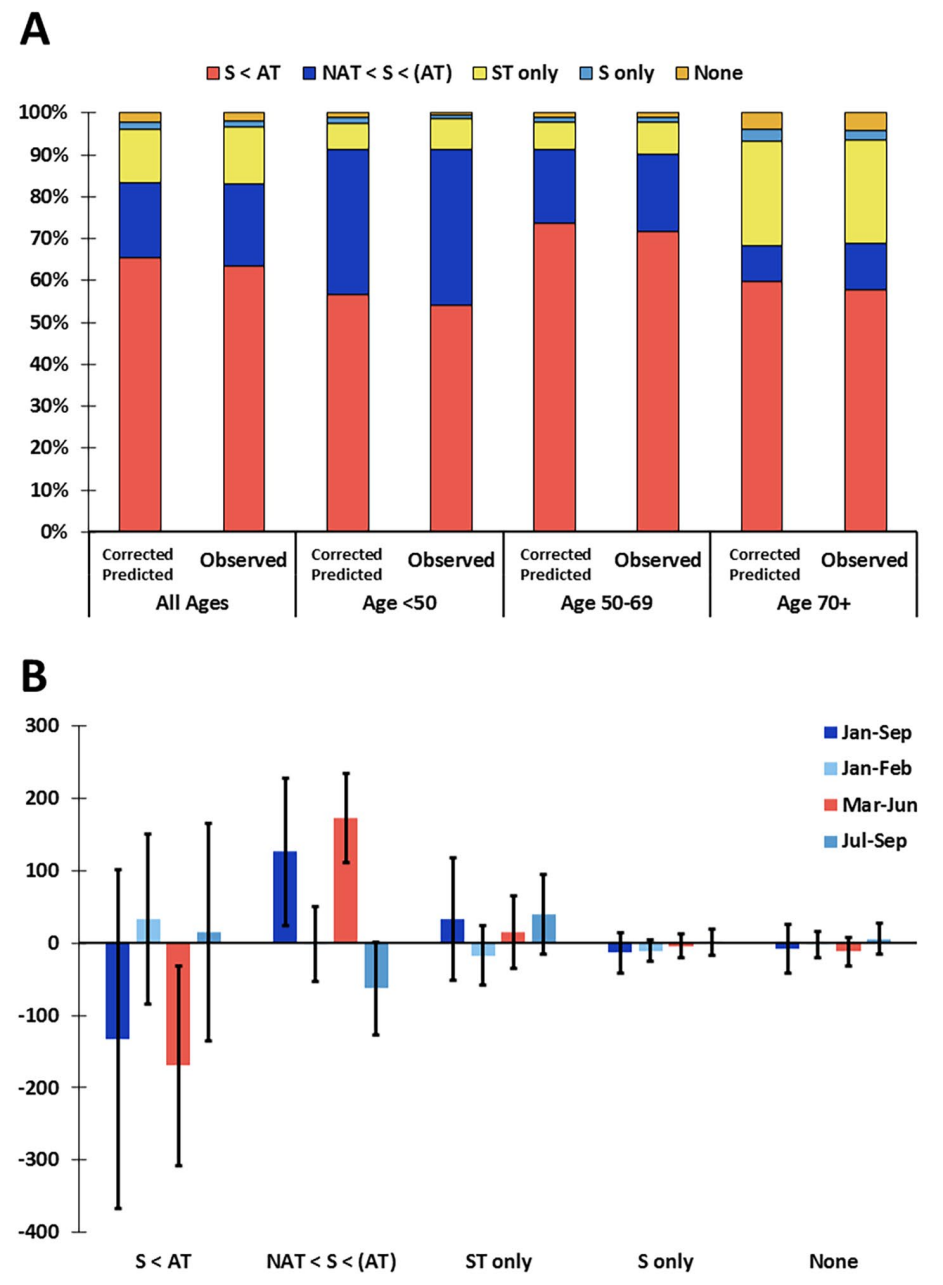
Treatment change from primary surgery to use of neoadjuvant systemic therapy in 2020. (**A**) Predicted (corrected for total decline in diagnoses) and observed distribution of treatment schemes in 2020 per age group indicating the percent of patients with invasive breast cancer diagnosed in January-September 2020 who received each treatment scheme. (**B**) Difference between the observed and the corrected-predicted number of patients with invasive breast cancer diagnosed in January-September 2020 by treatment scheme and by month of incidence. Error bars represent 95% confidence intervals. When the 95% confidence interval does not contain 0, the observed number of cases is significantly different from what would be predicted if the number of patients receiving each treatment scheme declined (or increased) proportional to the total decline (or increase) in cases relative to the reference period. S = surgery. ST = systemic treatment (hormonal therapy, targeted therapy, chemotherapy). NAT = neoadjuvant systemic therapy. AT = adjuvant therapy (systemic and/or radiotherapy). Patients with multiple invasive or breast tumors were excluded from this analysis

Overall, and in all age groups, there was an increased use of NAT + surgery, particularly significant for patients diagnosed in March-June 2020 (Fig. 3; Supplemental Fig. 7). For patients aged < 50 and 50–69 diagnosed in July-September there was a slight increase in the use of primary ST.

**Fig. 4:**
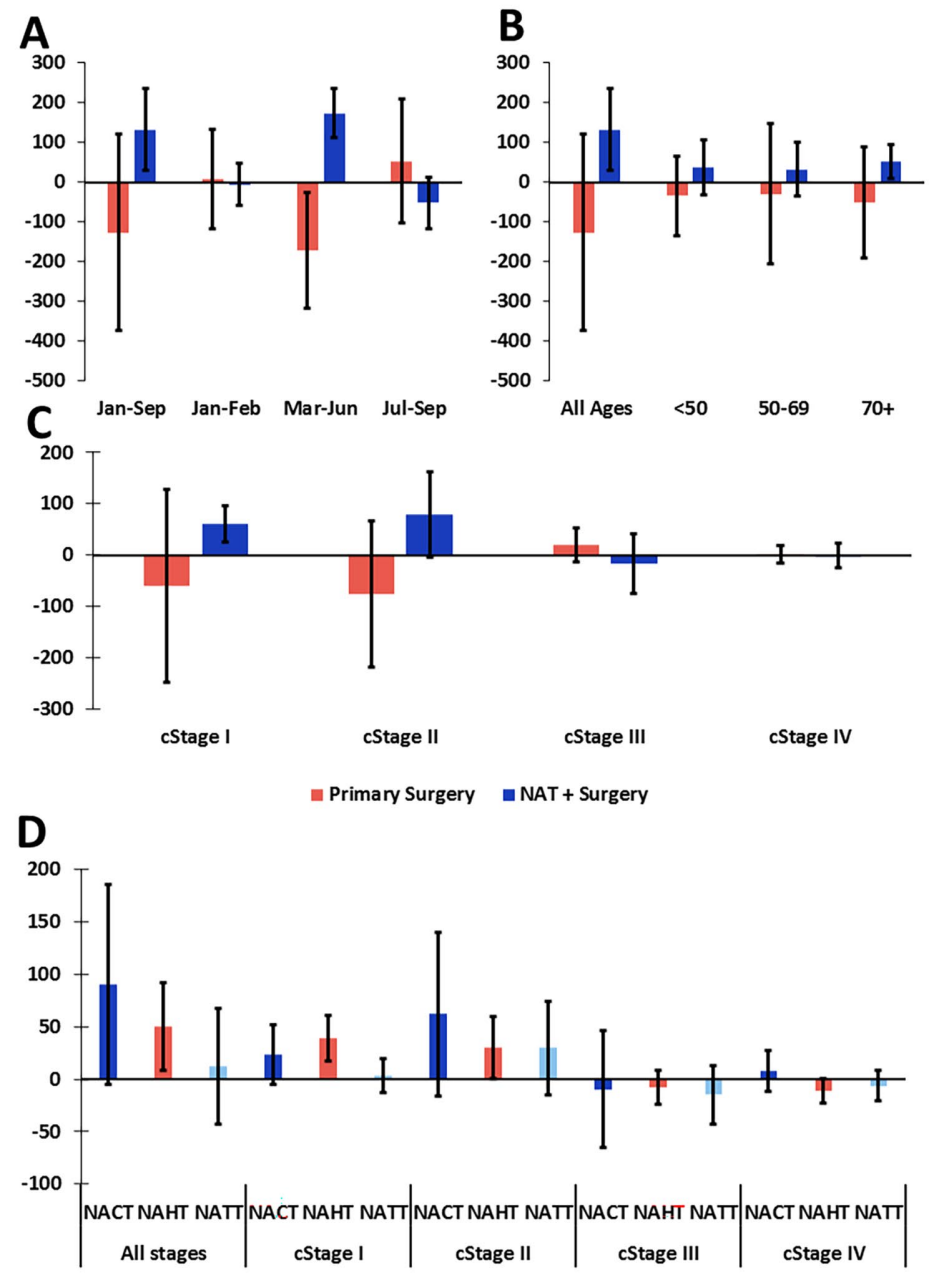
Increased use of neoadjuvant hormonal therapy in March-June 2020 in patients with lower stage tumors. Difference between the observed and predicted number of operated patients with invasive breast cancer diagnosed in January-September 2020 who received primary surgery or NAT followed by surgery, corrected for the total decline in diagnosis, by month of incidence (**A**), age group (**B**), and clinical stage (**C**). Difference between the observed and predicted number of patients with invasive breast cancer diagnosed in January-September 2020 who received each type of neoadjuvant systemic therapy, corrected for the total change in number of operated patients who received neoadjuvant systemic therapy, by clinical stage (**D**). NAT = neoadjuvant systemic therapy. NACT = neoadjuvant chemotherapy. NAHT = neoadjuvant hormonal therapy. NATT = neoadjuvant targeted therapy. Error bars represent 95% confidence intervals. When the 95% confidence interval does not contain 0, the observed number of cases is significantly different from what would be expected. Patients with multiple invasive or breast tumors were excluded from this analysis

Among operated patients, a significant shift was observed from primary surgery to NAT + surgery (Fig. 4A). This shift was particularly apparent among patients diagnosed in March-June 2020, in patients aged 70+, and in patients diagnosed with cStage I and II tumors (Fig. 4A-C). Among patients treated with NAT, the increase was only significant for neoadjuvant hormonal therapy (NAHT), and specifically in cStage I-II (Fig. 4D).

#### No increase in time to first treatment or duration of NAHT

**Fig. 5:**
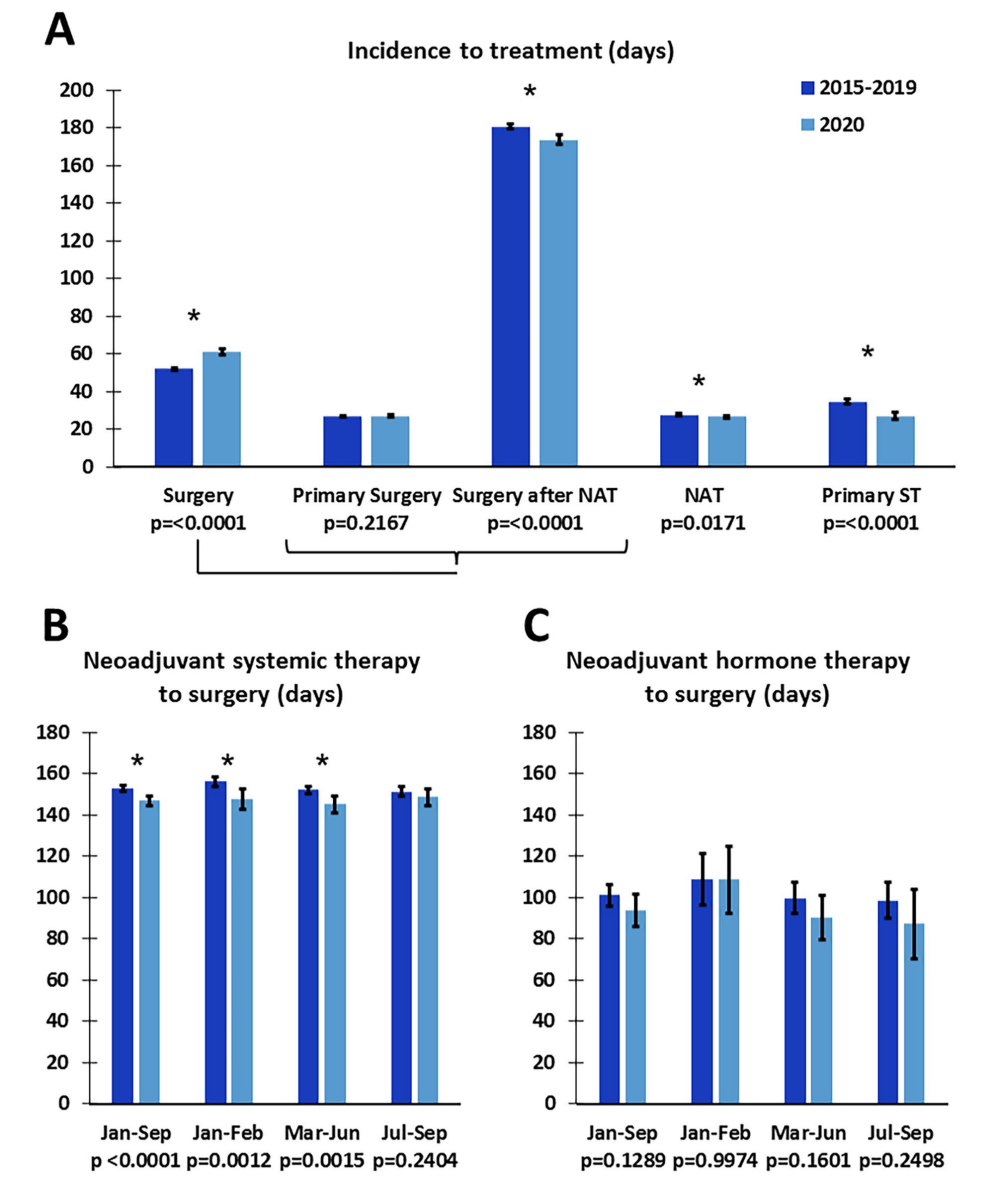
Shorter or unchanged average time to treatment for patients diagnosed from January-September 2020. (**A**) Average time (days) from incidence to the indicated treatment in 2020 versus 2015–2019 for patients with invasive breast cancer diagnosed in January-September. (**B**) Average time (days) from start of first neoadjuvant systemic therapy (chemotherapy, hormonal therapy or targeted therapy) to date of surgery, by month of incidence. (**C**) Average time (days) from start of neoadjuvant hormonal therapy to date of surgery for patients with invasive breast cancer diagnosed in January-September. Error bars represent 95% confidence intervals. * Indicates significant difference (*p* < 0.05) using a standard t-test or Welch’s t-test if variance was unequal according to Levene’s test for Homogeneity of Variance. Patients with multiple invasive or breast tumors were excluded from this analysis

The average number of days from incidence to start of treatment was compared between patients diagnosed in January-September 2015–2019 and January-September 2020 (Fig. 5). Among patients who received surgery without NAT there was no difference in time to surgery. Among patients who received NAT before surgery, the time from incidence to surgery was significantly shorter (Fig. 5A). The average time from incidence to start of NAT or start of primary ST was significantly shorter in 2020. Among all patients receiving NAT, the time from start of NAT to surgery was significantly shorter in 2020, while specifically for NAHT, there was no difference in time from start NAT to surgery (Fig. 5B&C). Similar trends were seen among patients receiving neoadjuvant chemotherapy (NACT) and neoadjuvant targeted therapy (NATT; Supplemental Fig. 8).

#### No change in type of breast surgery

Breast conserving surgery (BCS) was most common in all phases of the pandemic among the operated patients in January-September 2020 (Supplemental Fig. 9A). There was no significant difference in the proportion of patients receiving BCS versus mastectomy by clinical stage in 2020 compared to the 2015–2019 trends, except for proportionally more mastectomies among the patients diagnosed in March-June 2020 (Supplemental Fig. 9B&C).

#### Use of hypofractionated irradiation

In Belgium, a reimbursement code for hypofractionated breast irradiation in the context of COVID-19 was introduced in May 2020 [26]. In total, this code was billed for 8.2% of patients with in situ or invasive BC diagnosed in 2020 (data not shown). These patients were on average older, had a lower WHO performance score, had tumors with lower clinical stage, and received the irradiation in adjuvant setting.

## Discussion

### Delay in diagnosis but no stage migration in 2020

The BC incidence decline during the first wave of the COVID-19 pandemic in Belgium (March-June 2020) was primarily seen in cStage 0 and I, and in patients aged 50 and over. This finding can be explained by the temporary suspension of organized screening programs and non-essential healthcare, and is consistent with results from other countries showing reductions in screening volume and number of diagnosed BC [27–31]. In March-June 2020, we observed a higher proportion of tumors diagnosed with cStage II, however, the absolute number of cStage II tumors observed in 2020 was lower than the expected values. This finding indicates that – despite general healthcare restrictions - cStage II tumors were more likely to be diagnosed than lower stage tumors in 2020. Furthermore, for patients aged 70+, who are not invited for organized screening in Belgium, the decline in diagnosis impacted all tumor stages equally.

During the rebound period July-December 2020, even when considering annual increasing trends in incidence, higher than predicted absolute incidence numbers were observed indicating that missed diagnoses were partially recovered in the latter half of 2020. A similar rebound in missed diagnoses was not observed in population-based studies from The Netherlands or Scotland suggesting that Belgium was particularly successful in prioritizing oncologic care and restarting BC screening programs [30, 32]. Since both mentioned studies compared 2020 data to *average* values in previous years without accounting for increasing trends in BC incidence, the results in these reports should be interpreted with care.

There was no evidence for a general shift to a more advanced cStage of tumors diagnosed in July-December 2020. Contrarily, more cStage 0 tumors than predicted were diagnosed in this period, particularly in the screening age group, suggesting that much of the diagnostic recovery was due to effective resumption of the screening programs in Belgium [2]. However, 6% of female BC incidence was still outstanding at the end of 2020, which underlines the need to continue the monitoring of BC incidence and stage distributions in the subsequent years.

### Adaptations in care

The COVID-19 pandemic forced healthcare systems worldwide to implement priority-setting mechanisms in the management of cancer [33, 34]. For BC, the European Society for Medical Oncology (ESMO), among others, published specific recommendations including offering selected patients with luminal-like BC NAHT to avoid harm due to surgical delay [9–13, 35]. In Belgium, the overall proportion of patients receiving surgery (with/without NAT) did not change in 2020. In line with reports from numerous other countries and centers, our results showed a small shift from primary surgery to NAHT for patients diagnosed with early-stage BC [14, 29, 36–38]. Also, our findings showed no change in average pathological tumor diameter or nodal involvement between 2019 and 2020, both in the primary surgery population and in the NAT population separately. We therefore have the impression that patients were appropriately selected for this adapted approach. It is reassuring that on average the patients receiving primary surgery suffered no increased time to treatment, and the patients receiving NAT had a shorter time from incidence to start of NAT and to surgery, compared with previous years. In other countries, for example in The Netherlands, treatment delays were documented in the early weeks of the pandemic [29].

The recommendation to offer hypofractionated adjuvant radiotherapy to selected patients with BC to reduce hospital visits was documented for 8% of the patients in Belgium in 2020 [12, 13, 39].

### Still missing or postponed diagnoses

At the end of 2020 an estimated 660 BC (in situ/invasive) were still undiagnosed in Belgium [8]. These diagnoses were largely expected in the 70 + age group, a population not reached through organized screening programs in Belgium, and are predicted to mainly be cStage I tumors, which are less likely to progress rapidly [40]. Nevertheless, continuation of monitoring of the missed diagnoses and possible influence on stage at presentation during the next years is mandatory.

### Strengths and limitations of this study

Our study, unlike most studies that evaluated the impact of COVID-19 on BC incidence and stage in 2020, computes predicted values for 2020 based on the trends in incidence and stage over the previous 5 years to allow a reliable assessment of the impact of the pandemic [30, 32, 41–46]. In our opinion, the integration of these trends in the assessment of incidence, stage, and treatment of cancer during the pandemic in 2020 is of utmost relevance, knowing that cancer incidence is continuously evolving because of differences in age distribution, lifestyle, environmental factors, socioeconomic status, healthcare quality, screening programs etc [47].

Furthermore, our study used data from a highly complete population-based cancer registry combined with data from the national healthcare organizations covering all Belgian residents. The findings refer to the total BC population in Belgium and avoid possible bias introduced by changes in hospital choice or physician referral patterns in reaction to the COVID-19 pandemic.

Besides a thorough evaluation of possible changes in cStage of BC in 2020, our study also investigated the exact pathological tumor dimensions and nodal involvement through manual review of pathology records. Therefore, the clinical findings (cStage) were supplemented with pathological results.

Our study lacked valuable information such as the molecular subtype of BC, that plays an essential role in the priority-setting for management (e.g. patient selection for NAHT to safely postpone breast surgery), and the deprivation status of the patient, for which it was shown that the COVID-19 burden was higher in more deprived areas in Belgium [9–13, 35, 48]. Our study didn’t assess screening participation in detail, which other studies did document on and reported different associations between deprivation status, BC incidence and stage, and screening participation [30, 49–52]. Finally, regarding the completeness of the reimbursement data, details of adjuvant treatment and breast reconstruction were incompletely available for our study and remain to be assessed as more reimbursement data become available.

## Conclusions

The COVID-19 pandemic caused a decline in BC diagnoses in 2020 in Belgium, which was largely restricted to very early-stage breast tumors and patients aged 50 and older. The delay in diagnosis suffered during the first pandemic wave did not result in overall disease progression to more advanced tumors in the subsequent 6 months. Treatment adaptations observed in Belgium were in line with ESMO recommendations and were successful in prioritizing patients for surgery while preventing tumor progression in those with surgical delay. Continuation of monitoring of the missed diagnoses in 2020 and the possible influence on stage at presentation during the next years is desirable.

### Electronic supplementary material

Below is the link to the electronic supplementary material.

Supplementary Material 1

## Data Availability

The cancer cohort data used and analyzed during the study are available from the corresponding author upon reasonable request. The pseudonymized data can be provided within the secured environment of the BCR after having been guaranteed that the applicable GDPR regulations are applied.
