# Supplementary material for: Breast cancer incidence, stage distribution, and treatment shifts during the 2020 COVID-19 pandemic: a nationwide population-level study
Source: Arch Public Health. 2024 May 7;82:66. doi: 10.1186/s13690-024-01296-3 (PMC11075279; doi:10.1186/s13690-024-01296-3)
Supplement: Supplementary file 1 — Supplementary Material 1 [file 13690_2024_1296_MOESM1_ESM.docx]

**SUPPLEMENTAL TABLES AND FIGURES**

**Supplemental Table 1: Operated patients with invasive breast tumors and available pathological tumor dimensions & number of involved lymph nodes: comparison of exact average tumor dimensions (mm) and number of involved lymph nodes between incidence year 2019 and 2020, among patients who received primary surgery (A&B) and patients who received NAT followed by surgery (C&D).** NAT=Neoadjuvant treatment.

**
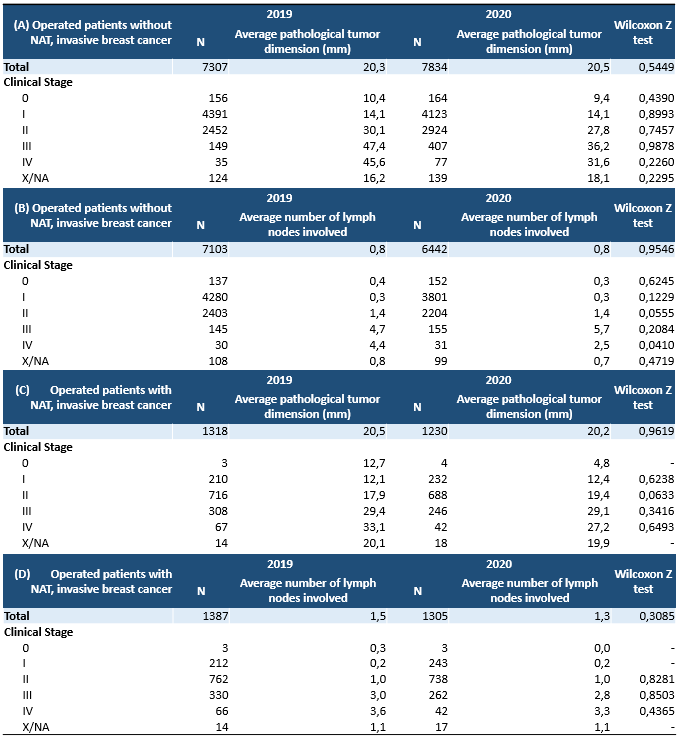
**

**Supplemental Table 2: Descriptive patient and tumor characteristics of all breast tumors (in situ and invasive) included in the study.** SD=standard deviation. IQR=inter-quartile range. IDC=invasive ductal carcinoma. ILC=invasive lobular carcinoma. DCIS=ductal carcinoma in situ. LCIS=lobular carcinoma in situ.

**Supplemental Table 3: Descriptive patient and tumor characteristics of all invasive breast tumors included in the study.** SD=standard deviation. IQR=inter-quartile range. IDC=invasive ductal carcinoma. ILC=invasive lobular carcinoma.

**Supplemental Table 4: Descriptive patient and tumor characteristics of all ductal carcinoma in situ breast tumors included in the study.** SD=standard deviation. IQR=inter-quartile range.

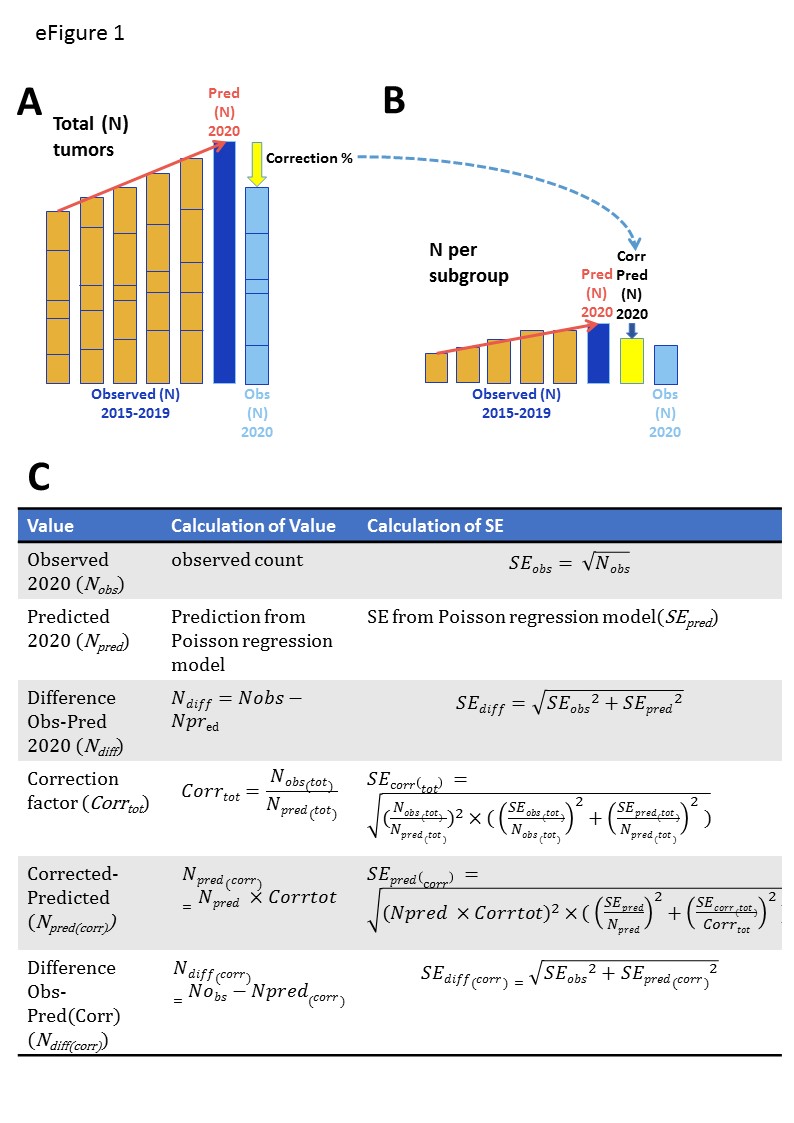


**Supplemental Figure 1: Method used to detect decline/increase in number (N) of diagnoses and disproportional decline/increase by subgroup (stage, treatment scheme etc.). (A)** Poisson count model was used to estimate the average yearly change in cancer diagnoses over the period 2015-2019 and extrapolated to 2020. **(B)** For each subgroup, the Poisson count model was applied independently to establish the predicted value for 2020. The predicted value for each subgroup was “corrected” for the percent decline/increase of the total group. Predicted values were compared to observed values for 2020. **(C)** Calculation of values and their standard errors.


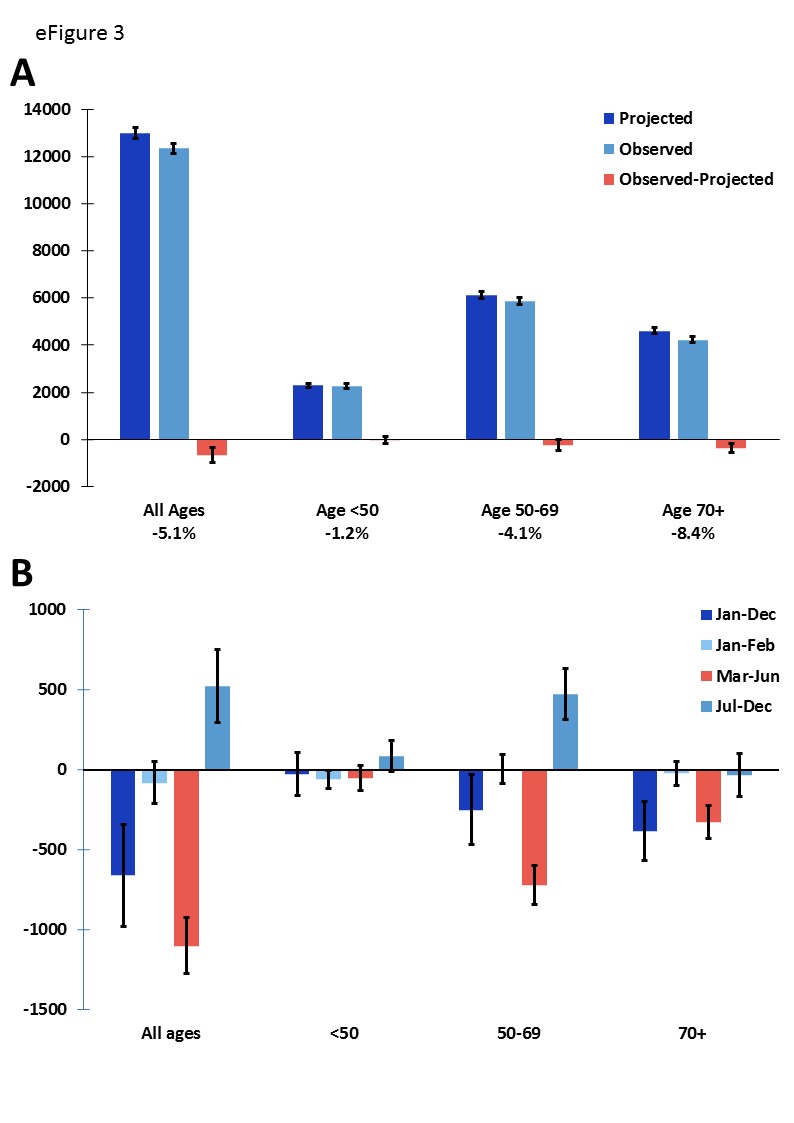


**Supplemental Figure 2: Largest decline in diagnosis for patients aged 70+. (A)** Predicted (dark blue), observed (light blue), and difference between predicted and observed (orange) number of cases of invasive and in situ breast cancer in 2020. **(B)** Difference between predicted and observed number of cases of invasive and in situ breast cancer in 2020 by age group and month of incidence. Error bars represent 95% confidence intervals. Differences between observed and predicted number of cases are significant if the 95% confidence interval does not contain 0.


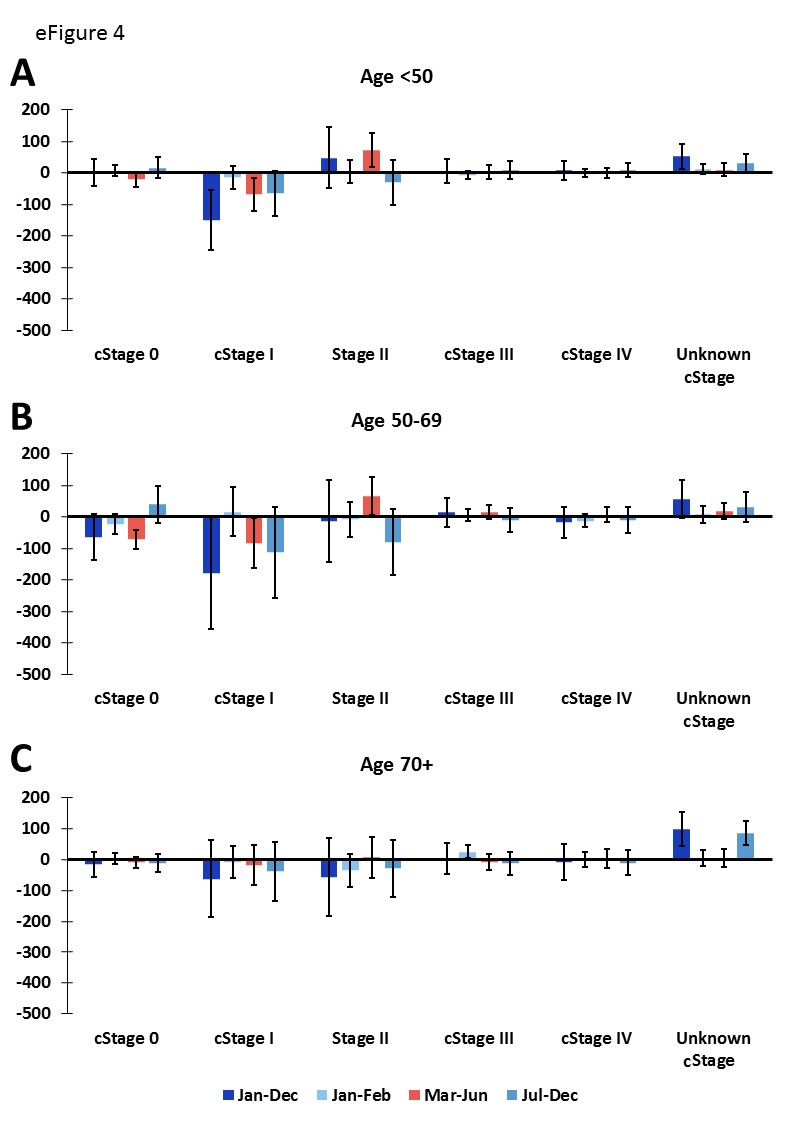


**Supplemental Figure 3: Impact on stage varied by age group.** Difference between the number of observed cases and the corrected-predicted number of cases of invasive or in situ breast cancer in 2020 by clinical stage and by month of incidence for patients aged <50 **(A)**, 50-69 **(B)**, and 70+ **(C)**. Error bars represent 95% confidence intervals. When the 95% confidence interval does not contain 0, the observed number of cases is significantly different from what would be predicted if the numbers in all stages declined (or increased) proportion to the total decline (or increase) in cases relative to the reference period.


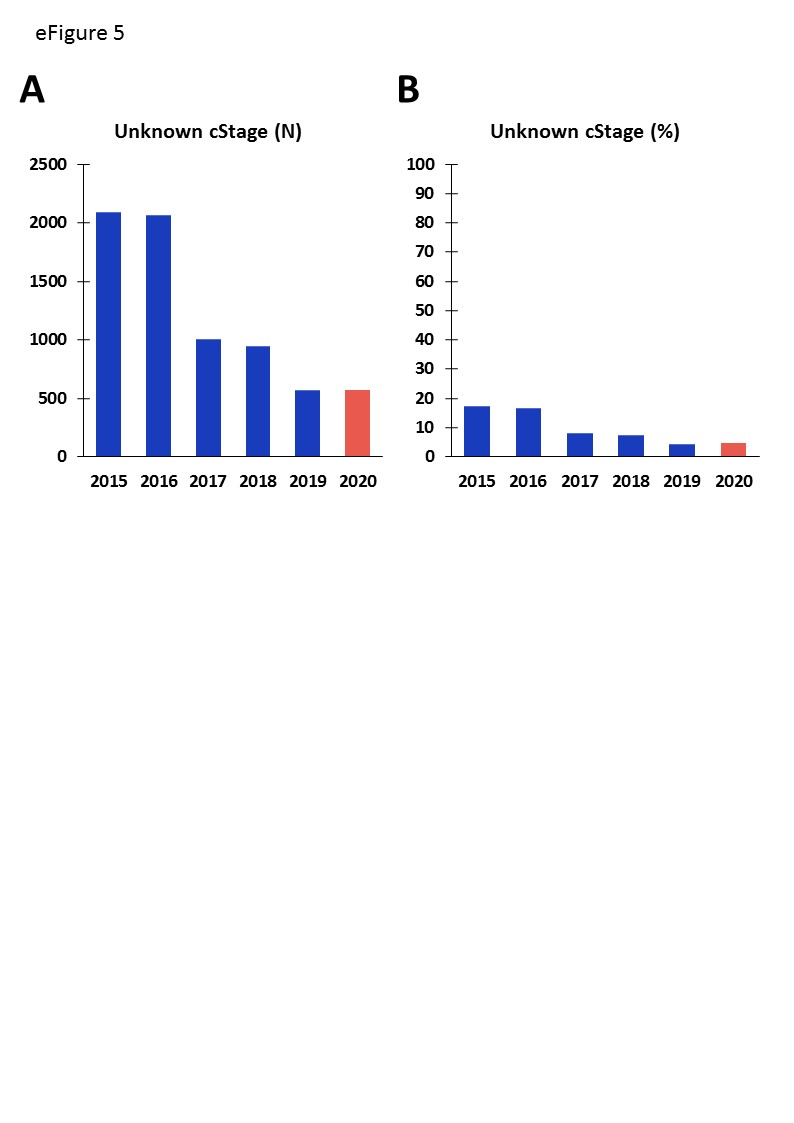


**Supplemental Figure 4: Unknown stage: trends for 2015-2020.** The number **(A)** and percent **(B)** of registered invasive or in situ breast tumors per year with unknown clinical stage.


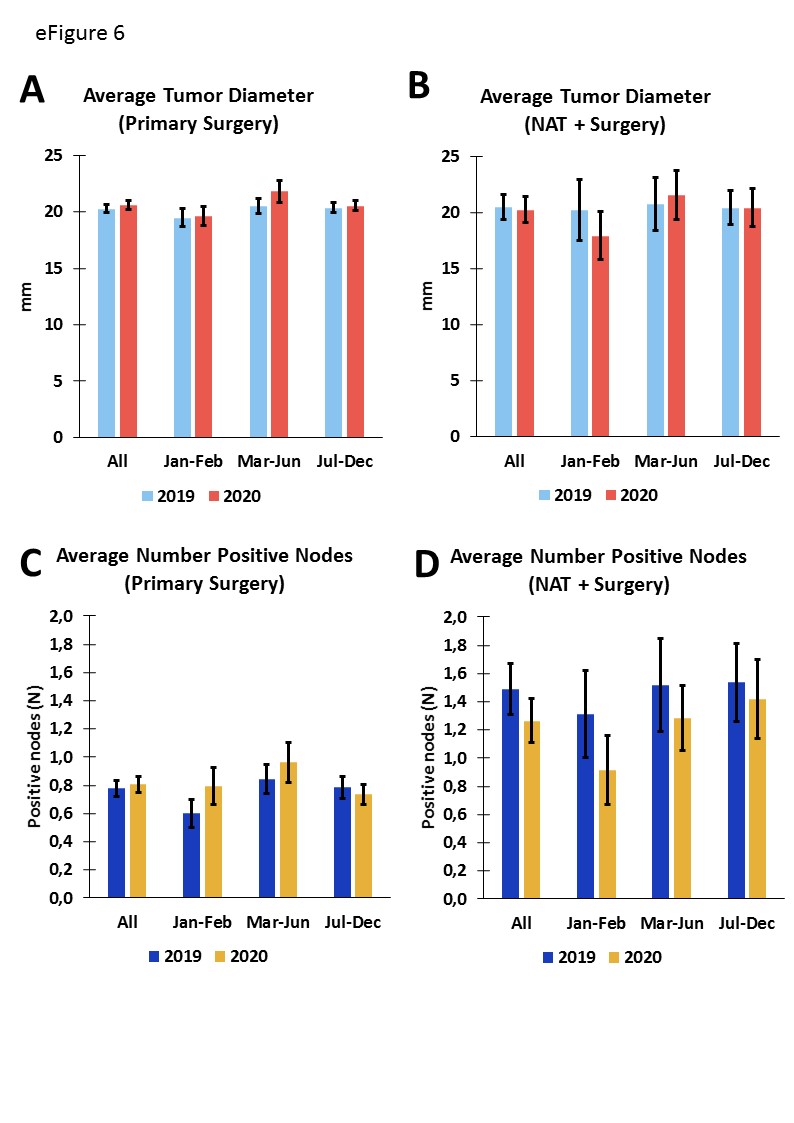


**Supplemental Figure 5: No difference in average tumor diameter or number of positive nodes for operated invasive tumors diagnosed in 2020 versus 2019.** Average tumor diameter (mm) **(A, B)** and average number of positive lymph nodes at resection **(C, D)** for operated invasive breast tumors in 2019 and 2020, among patients who received primary surgery **(A, C)** and patients who received NAT followed by surgery **(B, D)**. Error bars represent 95% confidence intervals. No differences were significant using a Wilcoxon Z Test. NAT=neoadjuvant systemic therapy.


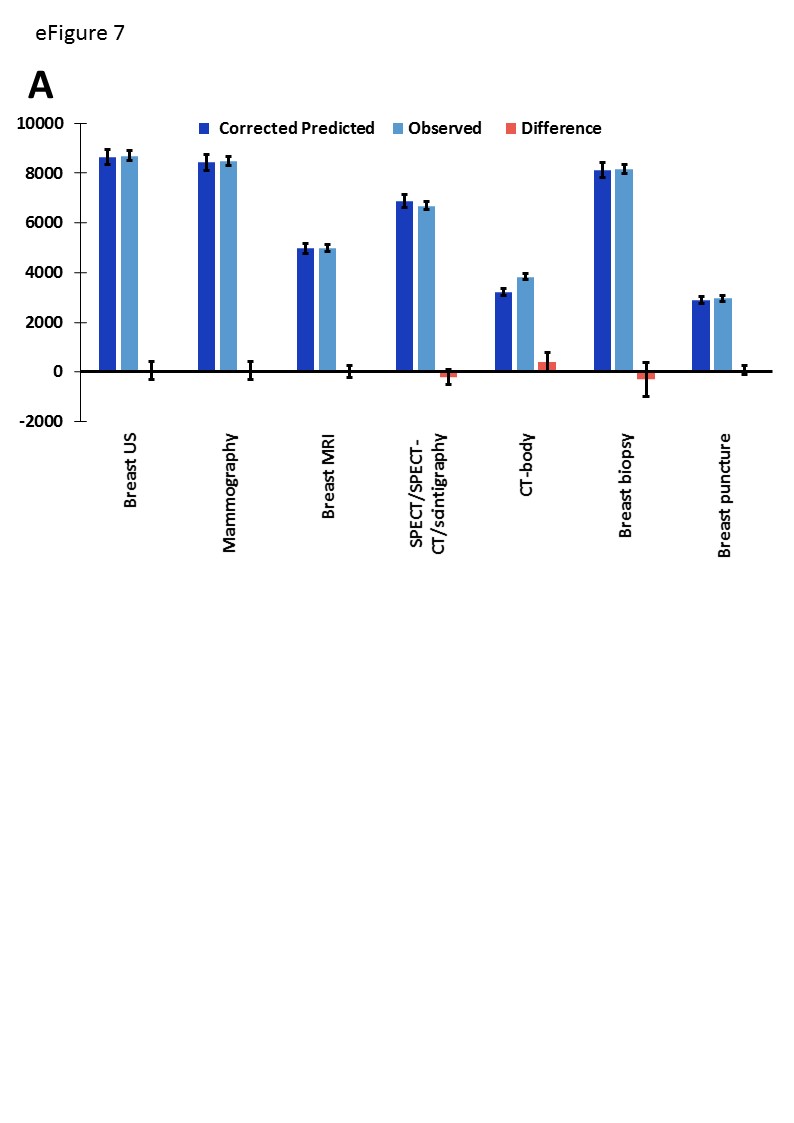


**Supplemental Figure 6: Diagnostic procedures for breast cancer in 2020. (A)** Predicted corrected for total decline in diagnoses (dark blue), observed (light blue) and difference between observed and predicted-corrected (orange) number of patients with invasive breast cancer undergoing the specified diagnostic procedures from 90 days before to 90 days after incidence in 2020.


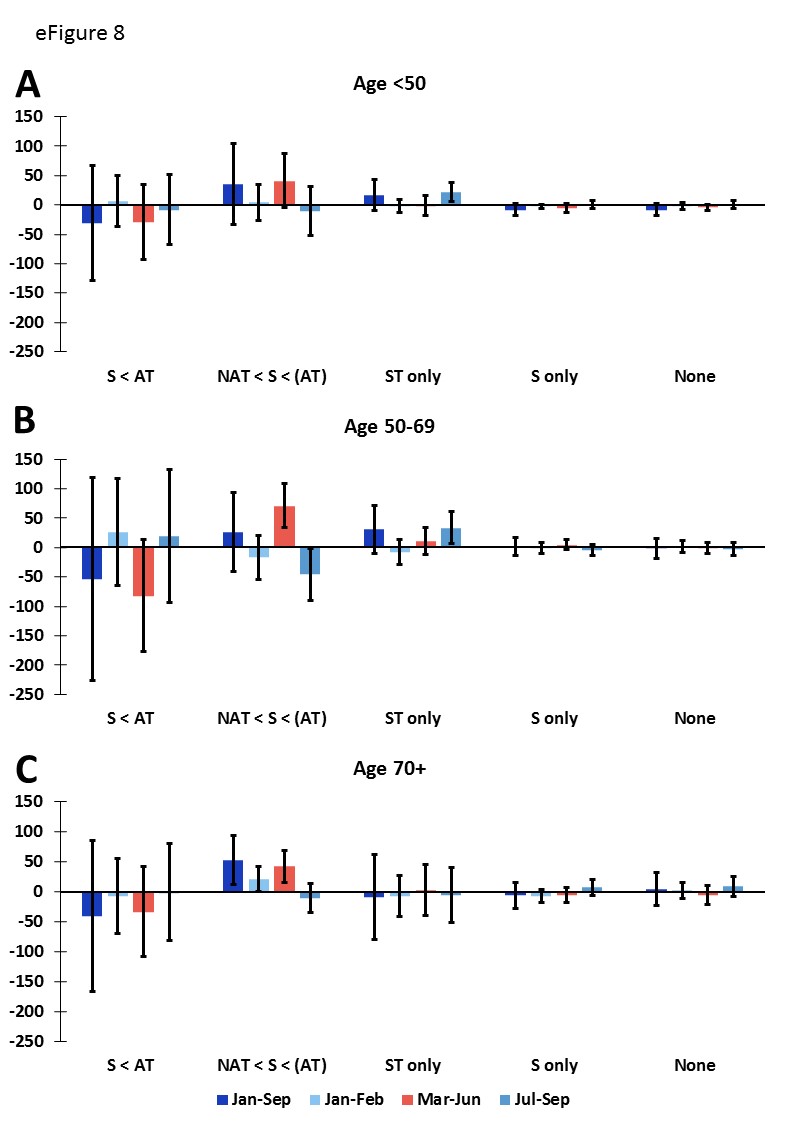


**Supplemental Figure 7: Increased neoadjuvant therapy use for invasive breast cancers diagnosed in March-June in all age groups.** Difference between the observed and the corrected-predicted number of patients aged <50 **(A)**, 50-69 **(B)**, and 70+ **(C)** with invasive breast cancer diagnosed in January-September 2020 by treatment scheme and by month of incidence. Error bars represent 95% confidence intervals. When the 95% confidence interval does not contain 0, the observed number of cases is significantly different from what would be predicted if the number of patients receiving each treatment scheme declined (or increased) proportion to the total decline (or increase) in cases relative to the reference period. S=surgery. ST=systemic treatment (hormonal therapy, targeted therapy, chemotherapy). NAT=neoadjuvant systemic therapy. AT=adjuvant therapy (systemic and/or radiotherapy). Patients with multiple invasive or breast tumors were excluded from this analysis.


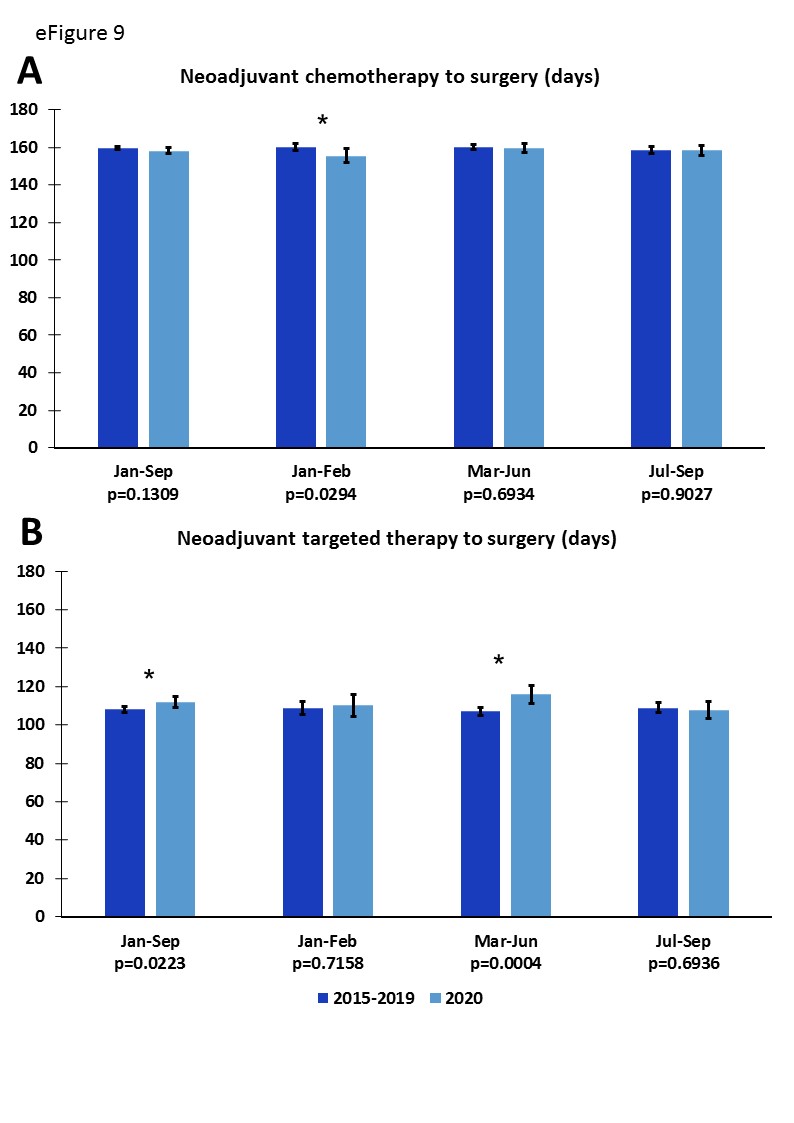


**Supplemental Figure 8: Average time (days) from start of neoadjuvant therapy to surgery in 2020 versus 2015-2019.** Average time (days) from start of neoadjuvant chemotherapy **(A)** or neoadjuvant targeted therapy **(B)** to date of surgery for patients with invasive breast cancer diagnosed in January-September. Error bars represent 95% confidence intervals. * Indicates significant difference (p<0.05) on standard t-test or Welch’s t-test if variance was unequal according to Levene’s test for Homogeneity of Variance. Patients with multiple invasive or breast tumors were excluded from this analysis.


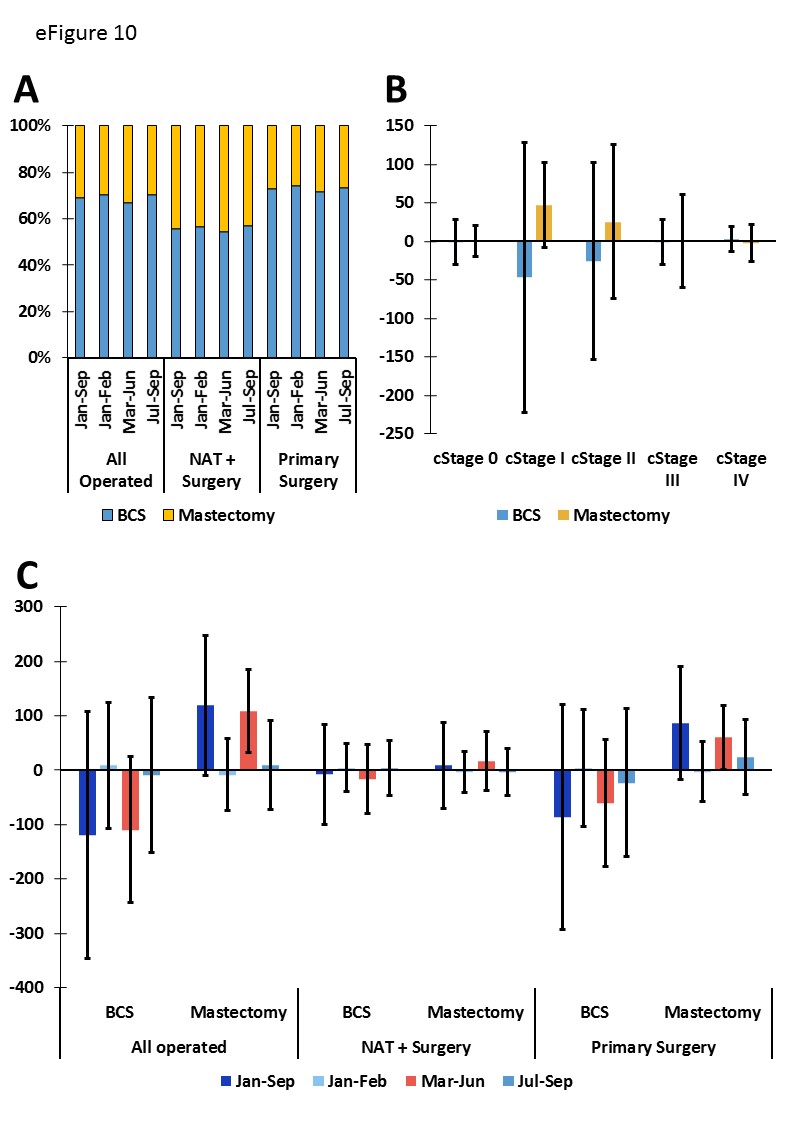


**Supplemental Figure 9: Increased use of mastectomy among patients with invasive breast cancer diagnosed in March-June 2020 who underwent primary surgery.** Observed proportions of patients receiving BCS (blue) or mastectomy (yellow) in 2020 **(A)**. Difference between the observed and the corrected-predicted number of patients receiving BCS or mastectomy in 2020 by clinical stage **(B)**, and by NAT status and month of diagnosis **(C)**. Error bars represent 95% confidence intervals. When the 95% confidence interval does not contain 0, the observed number of cases is significantly different from what would be predicted if the number of patients receiving each surgery type declined (or increased) proportion to the total decline (or increase) in number of patients receiving surgery relative to the reference period. BCS=Breast-conserving surgery. NAT=neoadjuvant systemic therapy. Patients with multiple invasive or breast tumors were excluded from this analysis.
